# A Subset of Circulating Blood Mycobacteria-Specific CD4 T Cells Can Predict the Time to *Mycobacterium tuberculosis* Sputum Culture Conversion

**DOI:** 10.1371/journal.pone.0102178

**Published:** 2014-07-21

**Authors:** Catherine Riou, Clive M. Gray, Masixole Lugongolo, Thabisile Gwala, Agano Kiravu, Pamela Deniso, Lynsey Stewart-Isherwood, Shaheed Vally Omar, Martin P. Grobusch, Gerrit Coetzee, Francesca Conradie, Nazir Ismail, Gilla Kaplan, Dorothy Fallows

**Affiliations:** 1 Division of Immunology, Institute of Infectious Disease and Molecular Medicine and National Health Laboratory Services, University of Cape Town, Cape Town, South Africa; 2 National Institute for Communicable Diseases, National Health Laboratory Services, Johannesburg, South Africa; 3 Right to Care and the Clinical HIV Research Unit, University of the Witwatersrand, Johannesburg, South Africa; 4 Center of Tropical Medicine and Travel Medicine, Department of Infectious Diseases, Division of Internal Medicine, Academic Medical Center, University of Amsterdam, Amsterdam, The Netherlands; 5 Laboratory of Mycobacterial Immunity and Pathogenesis, Public Health Research Institute at the International Center for Public Health, Newark, New Jersey, United States of America; National Institute for Infectious Diseases (L. Spallanzani), Italy

## Abstract

We investigated 18 HIV-negative patients with MDR-TB for *M. tuberculosis* (Mtb)- and PPD-specific CD4 T cell responses and followed them over 6 months of drug therapy. Twelve of these patients were sputum culture (SC) positive and six patients were SC negative upon enrollment. Our aim was to identify a subset of mycobacteria-specific CD4 T cells that would predict time to culture conversion. The total frequency of mycobacteria-specific CD4 T cells at baseline could not distinguish patients showing positive or negative SC. However, a greater proportion of late-differentiated (LD) Mtb- and PPD-specific memory CD4 T cells was found in SC positive patients than in those who were SC negative (p = 0.004 and p = 0.0012, respectively). Similarly, a higher co-expression of HLA-DR^+^Ki67^+^ on Mtb- and PPD-specific CD4 T cells could also discriminate between sputum SC positive versus SC negative (p = 0.004 and p = 0.001, respectively). Receiver operating characteristic (ROC) analysis revealed that baseline levels of Ki67^+^HLA-DR^+^ Mtb- and PPD-specific CD4 T cells were predictive of the time to sputum culture conversion, with area-under-the-curve of 0.8 (p = 0.027). Upon treatment, there was a significant decline of these Ki67^+^HLA-DR^+^ T cell populations in the first 2 months, with a progressive increase in mycobacteria-specific polyfunctional IFNγ^+^IL2^+^TNFα^+^ CD4 T cells over 6 months. Thus, a subset of activated and proliferating mycobacterial-specific CD4 T cells (Ki67^+^HLA-DR^+^) may provide a valuable marker in peripheral blood that predicts time to sputum culture conversion in TB patients at the start of treatment.

## Introduction

The tuberculosis (TB) epidemic in many parts of the world has been greatly exacerbated in recent years, not only by the HIV co-epidemic, but also by the rise in multidrug resistant (MDR) strains of *M. tuberculosis* (Mtb). MDR-TB is defined by resistance to rifampicin (RIF) and isoniazid (INH), the two most effective drugs against TB and the backbone of standard short-course therapy [1], [2]. The quest for new pharmaceuticals to combat both drug susceptible and resistant TB and expand treatment options for patients with MDR-TB is a major challenge. At present, the success of TB therapy is established by the risk of relapse within the first 2 years after treatment, which necessitates long clinical trials and extended follow-up of patients. Thus, to support clinical trials and improve case management, early predictors of clinical outcome that can serve as interim indicators of treatment response are needed. The only currently accepted interim indicators are sputum culture conversion after 2 months of standard therapy and time to culture positivity at the start of treatment, which provides an indicator of bacillary load [3]–[5]. However, culture-based methods require 6–8 weeks for a result and are only appropriate for patients who are sputum culture positive at baseline. To address this clinical need, as well as to shorten the time required for clinical trials of new TB drugs in the pipeline, extensive efforts to discover early biomarkers of response to TB treatment are currently underway [6]–[9].

The evaluation of candidate host immune biomarkers is a particularly active area of research, which additionally can contribute to our general understanding of the pathogenesis of TB disease [10]. A number of investigators have examined serodiagnostic markers in patients before and during TB treatment, including inflammatory molecules, cytokines and chemokines, as well as antibodies against Mtb proteins [10]–[12] and the host blood transcriptome [13], [14]. Others have assessed various immune cell populations in peripheral blood and bronchoalveolar lavage [10], [15], [16]. Although several groups have described associations between specific markers of T cell activation/function and outcome of TB treatment, there is, as yet, no consensus in the field about the most promising candidates. Importantly, many of the reported studies have used cross-sectional designs to identify candidate biomarkers that can differentiate patients with active disease from individuals who have successfully completed TB treatment or have latent TB infection, rather than following patients prospectively. Moreover, few studies have examined the generalizability of candidate biomarkers for use in monitoring patients during treatment of MDR-TB [17]. This is an important question, as rising rates of MDR-TB are increasingly hampering TB control, particularly in regions with high prevalence of TB and HIV [18] [19]. Moreover, the specific issues associated with conducting randomized controlled trials of drug regimens in MDR-TB patients support the value of investigating interim markers of response to treatment in this population [20].

As part of a prospective cohort study to investigate host factors associated with delayed response to treatment and treatment failure in patients with MDR-TB, we have carried out a pilot study to examine T cell markers in 18 patients. Our goal was to correlate specific T cell subsets in blood with bacillary (antigen) load, as indicated by sputum culture (SC) positivity, and to investigate changes in the relative frequencies and phenotypic characteristics of mycobacteria-specific CD4 T cell subsets in patients responding to TB treatment. Our results show that TB patients have highly activated mycobacteria-specific CD4+ T cells, which become less activated as patients respond to chemotherapy. Validation of our findings in a larger group of patients, including those with drug susceptible TB, may identify a host cellular phenotype that can provide a useful early marker predictive of time to culture conversion.

## Materials and Methods

### Study participants and sample collection

Participants (n = 18) were recruited at Sizwe Hospital, the central MDR-TB referral hospital in Gauteng Province, South Africa. The study cohort had a median age of 39 years (IQR: 26–51 years) and a sex ratio of 13∶5 male:female. All enrolled patients were HIV negative. TB diagnosis was based on sputum culture; diagnosis of MDR was based on phenotypic drug susceptibility testing (DST by Bactec MGIT 960, Beckton Dickinson, Baltimore, MD) and/or genotypic resistance by PCR (Line Probe Assay, Hain Lifesciences). All study participants were diagnosed with culture positive MDR-TB based on sputum specimens obtained at a median of 57 days (IQR: 33–99 days) prior to admission to Sizwe Hospital (Table S1). As part of the admission process, routine chest X-ray (CXR) was performed. CXR films were reviewed by physicians (who were not study investigators) and scored for cavitation (no, single or multiple cavities; <2, 2–4 or >4 cm diameter) and extent of disease (0, <50% or ≥50% of area affected) in six zones, defined by the mediastinum and horizontal lines through the 2^nd^ and 4^th^ anterior rib shadows [21]. Nine patients showed cavitation and all had evidence of TB disease by CXR (Table S1), with the exception of #16, who showed no cavitation and no significant signs of disease except for a possible right parahilar mass.

Of the 18 patients enrolled, 15 had a prior history of TB, two of whom (#3 and #11) had previously been treated for MDR-TB and were receiving second line drugs at the time of admission to Sizwe Hospital. Thirteen patients were receiving short-course TB chemotherapy (with or without streptomycin) at the time of admission; two (#9 and #12) had been diagnosed with primary MDR-TB after initiating short-course therapy for drug susceptible TB and 11 were admitted after failing short-course therapy. Three patients had been admitted with primary MDR-TB and were TB treatment naïve (Table 1). Upon admission to Sizwe, patients were initiated on a standardized MDR-TB drug regimen, consisting of kanamycin, ofloxacin, ethionamide, terizodone and pyrazinamide [21]. This regimen was modified for individual patients on the basis of DST results indicating resistance to any of these drugs and for those with prior history of treatment for MDR-TB. Additional adjustments in regimens were made, as needed, for patients showing adverse reactions to specific drugs during the course of treatment (Table S1).

**Table 1:** Microbiological characteristics of patients enrolled into the study showing pre-admission and on-admission (visit 0) AFB smear, time between smears and time to first sputum culture negative, culture conversion and which antigen specificities were detectable in the ICS assay (% responders).

PID	Age	Sex	Prior TB Episodes%	MDR Classification§	On TB Treatment at Admission		Admission AFB^1^ smear	Admission Sputum Culture (SC)	Time to culture conversion (days)*	Mtb	PPD	Mito^2^
1	51	M	Y	2	Y	b	neg	pos	32	Y	Y	Y
2	50	M	Y	2	N	-	+	pos	33	N	Y	Y
3	28	M	Y	9	Y	c	neg	pos	34#	N	Y	Y
4	24	M	Y	2	Y	a	+++	pos	65	Y	Y	Y
5	26	F	Y	2	y	a	+++	pos	65	Y	Y	Y
6	26	M	Y	2	Y	b	+++	pos	92	Y	Y	Y
7	44	F	Y	2	Y	b	+	pos	98	Y	Y	Y
8	51	M	Y	2	N	-	++	pos	114	Y	Y	Y
9	23	M	Y	6	Y	a	+++	pos	116	Y	Y	Y
10	39	M	Y	2	Y	a	+++	pos	120	Y	Y	Y
11	38	M	Y	4	Y	d	+++	pos	126	N	Y	Y
12	35	F	Y	6	Y	a	+	pos	177	N	Y	Y
13	22	M	N	6	N	-	neg	neg	-	N	Y	Y
14	55	F	Y	2	Y	a	neg	neg	-	Y	Y	Y
15	58	M	Y	2	Y	a	neg	neg	-	Y	Y	Y
16	50	F	N	6	Y	a	neg	neg	-	N	Y	Y
17	39	M	Y	2	Y	b	neg	neg	-	Y	Y	Y
18	62	M	N	6	Y	a	neg	neg	-	Y	Y	Y
									% responders	67%	100%	100%

% Y =  yes; N =  no.

§ MDR Classification: 2, New MDR after TB treatment failure; 4, MDR after TB default; 6, Primary MDR; 9, MDR 2nd episode.

1 Acid Fast Bacilli (intensity score: +).

2 Mitogen (QuantiFERON-TB Gold).

*culture conversion calculated as the interval between the date of admission/start of MDR regimen to date of the first of 2 consecutive negative cultures at least 30 days apart.

# based on first negative sputum culture, no follow up sputum culture results recorded.

a RIF/INH/EMB/PZA (Reg 1).

b Reg 1 plus Streptomycin.

c Reg 1 plus Ofloxacin/Ethionamide/Streptomycin.

d PAS/Terizodone/High dose INH/Kanamycin/Clarithromycin.

Blood samples for the study were collected at enrollment upon admission to Sizwe Hospital and then at 2, 4 and 6 months after initiating MDR-TB therapy. Peripheral blood was obtained in sodium heparin Vacutainer tubes (BD Biosciences, San Diego, CA, USA).

### Ethics Statement

All study participants gave written, informed consent for the study, which was approved by the Research Ethics Committees of the Universities of the Witwatersrand (HREC protocol M090357) and Cape Town and the Institutional Review Board of the University of Medicine and Dentistry of New Jersey.

### Reagents and antibodies

QuantiFERON-TB Gold tubes consisting of a Negative control (Nil antigen); Mtb tube (containing of a cocktail of ESAT-6, CFP-10 and TB7.7(p4) peptides); Mitogen (Mito) tube, (containing phytohaemagglutinin (PHA) as the positive control) were obtained from Cellestis Inc. (Valencia, CA, USA) and used in the whole blood intracellular cytokine (ICS) assay. Tuberculin purified protein derivative (PPD) was obtained from the Staten Serum Institute (Copenhagen, Denmark) and used as a separate stimulus at 10 µg/ml in the whole blood ICS assay. The following fluorescently conjugated mAbs were used in this study: anti-CD8 V500 (RPA-T8), anti–IFNγ Alexa Fluor 700 (B27), anti-TNFα PEcy7 (MAb11), anti-Ki-67 FITC (B56) and anti-IL2 PE (MQ1-17H12), all from BD Biosciences (San Diego, CA, USA); anti-CD3 Brilliant Violet 650 (Okt3) from Biolegend (San Diego, CA, USA) anti-CD4 PEcy5.5 (S3.5) from Invitrogen (Grand Island, NY, USA), anti-CD45RA ECD (2H4) from Beckman Coulter (Brea, CA, USA); anti-CD27 PEcy5 (323) and anti-HLA-DR APC-eFluor780 (LN3) from eBioscience (Wembley, UK).

### 12 hour-Whole blood ICS assay

Within a maximum of 45 min after blood collection, 1 ml of sodium heparinized blood was incubated with PPD (10 µg/ml) or added to the QuantiFERON-TB Gold tubes in the presence of anti-CD28 (1 µg/ml, BD Pharmingen) and anti-CD49d (1 µg/ml, BD Pharmingen) for 7 hrs at 37°C. Blood incubated in the QuantiFERON-Nil Gold tube served as a negative control, and blood incubated in the QuantiFERON-Mitogen Gold tube as a positive control. After 7 hrs, brefeldin A (10 µg/ml; Sigma-Aldrich, Dorset, UK) was added, and the incubation continued for an additional 5 hrs [22]. Following the 12 hrs incubation, 2 mM EDTA was added for 10 min, then RBC were lysed and white blood cells were fixed with FACS Lysing Solution (BD Biosciences), followed by cryopreservation in freezing medium containing 10% DMSO and stored in liquid nitrogen until use. Cryopreserved cells were thawed, washed in PBS, permeabilized with Perm/Wash solution (BD Biosciences). Cells were stained with a panel of conjugated mAbs, described above, for 45 min at 4°C, washed in Perm/Wash Buffer (BD Biosciences), and resuspended in PBS containing 1% formaldehyde, prior to acquisition on an LSRII flow cytometer (BD Biosciences). At least 1.5 million total events were acquired for each sample.

### Flow cytometry data analysis

Multiple-parameter flow cytometry data were analyzed using FlowJo software (v9.5.3; Tree star Inc., Ashland, OR, USA). The gating strategy is presented in Figure S1. Combinations of cytokine-producing cells were determined using Boolean gating in FlowJo, followed by further analysis using Pestle v1.6.2 and Spice v5.3 (Dr. Mario Roederer, Vaccine Research Center, National Institute of Allergy and Infectious Diseases, National Institutes of Health, Bethesda, MD, USA). Cytokine responses were considered positive when the frequency of total cytokine-producing CD4 T cells was two times greater than the frequency of total cytokine-producing CD4 T cells in the negative control and greater than 0.05%. Background cytokine production in the negative control of ICS assays was subtracted from each antigen-stimulated condition. For phenotypic and polyfunctional analysis, only individuals with positive T cell responses to Mtb antigens (n = 12/18) and PPD (n = 18/18) were included, as shown in Table 1.

### Bacteriological assessment of sputum acid-fast bacilli smear and sputum culture

Sputum culture and MGIT DST were performed at the time of admission to Sizwe Hospital and monthly thereafter, as part of routine care to monitor the progress of treatment. In addition, sputum smear microscopy was performed regularly to monitor the presence of acid-fast bacilli (AFB) under direct fluorescent microscopy by auramine stain. The results of routine microbiological testing were utilized for this study. Sputum culture conversion was defined as two consecutive negative sputum culture results, separated by at least 30 days, with no subsequent culture positive results. Time to culture conversion was determined from the interval between the initiation of treatment for MDR-TB at Sizwe Hospital and the date of collection of the sputum specimen that yielded the first negative culture result, in accordance with the South African National Guidelines for Treatment of MDR-TB [21]. For the purposes of this study, the time of admission to Sizwe Hospital for treatment of MDR-TB and enrollment into the study are defined as baseline.

### Statistical Analysis

Statistical testing was performed using GraphPad Prism v5.0a software (GraphPad Prism software version 5 (Software MacKiev, GraphPad, San Diego, CA). Data were expressed as median values and analyzed by the use of nonparametric statistics. Statistical significance was determined using Mann-Whitney, Wilcoxon Paired *t* test, or Kruskal-Wallis ANOVA using Dunn's test for multiple comparisons. All tests were two-tailed, and a value of p<0.05 was considered statistically significant. Statistical analysis of T cell multi-cytokine expression differences between groups, represented in pie-chart format, was made using a non-parametric partial permutation method within SPICE [23]. The relationship between the proportions of activated CD4 T cells and time to sputum culture negative was assessed by either Pearson or Spearman rank correlations, depending on whether data was normally distributed and passed the Shapiro-Wilk normality test. Receiver operator characteristics (ROC) and area under the curve (AUC) were used to assess the predictive nature of CD4 subsets with time to culture conversion.

## Results

### Cohort Characteristics

For analysis, patients were assigned into two groups based on sputum culture (SC) results at the time of initiating treatment for MDR-TB at Sizwe Hospital (baseline): SC positive and SC negative. Table 1 shows the microbiological characteristics and TB history of each patient prior to and at the time of hospital admission and enrollment. Six patients were negative for AFB smear and Mtb culture (SC negative) at baseline, but all were SC positive at a median of 56 (IQR: 42–72) days prior to admission (Table S1). Three of these patients (50%) had a prior history of TB, 5 (83%) were on treatment for drug susceptible TB at the time of admission and one was TB treatment naïve. These patients had a median CXR disease score of 5.50 (IQR: 1.00–6.00), and one (17%) had cavitary disease at baseline. All of the 12 patients who were SC positive on admission had a prior history of TB; eight (67%) were on treatment for drug susceptible TB, two (17%) were receiving second line drugs and two (17%) were not on TB treatment. Sputum smear results at baseline ranged from negative to AFB+++ (Table S1). Among these patients, the median CXR disease score was 7.00 (IQR: 7.00–9.00), and nine (75%) had cavitary disease on admission. Thus, in general, the patients who were SC positive at baseline had a more extensive history of TB and showed more severe clinical signs of disease. While the majority of patients in both groups were on TB chemotherapy at the start of the study, the SC negative patients were apparently more responsive to the suboptimal drug regimen, leading to lower bacillary load at baseline. Among the 12 SC positive patients, the time to first negative sputum Mtb culture ranged from 32–177 days (Table 1). One patient (#3) was discharged after the first negative culture result and did not have any further results recorded, so that culture conversion could not be confirmed. At baseline, all patients exhibited a positive response to PPD and mitogen, as measured by whole blood intracellular cytokine staining, and 12/18 showed detectable responses to Mtb antigens.

### Frequencies of Mtb- and PPD-specific CD4 T cells cannot distinguish patients who are SC positive and SC negative at baseline

**Figure 1:**
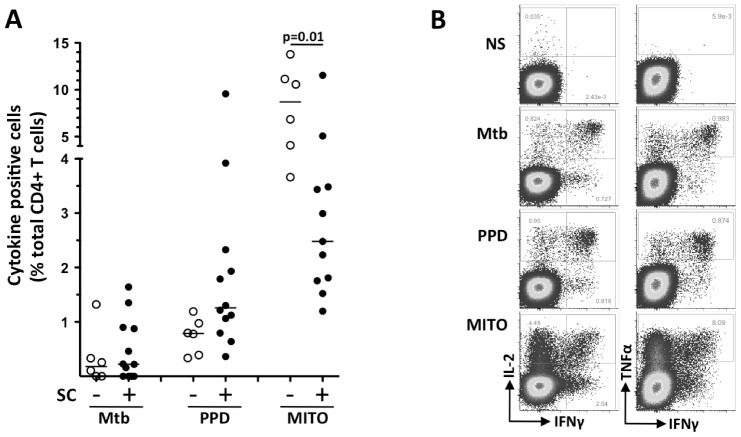
Comparison of the frequencies of Mtb, PPD and mitogen responsive CD4 T cells at baseline between patients who were sputum culture (SC) negative or positive. (A) The magnitude of antigen-specific CD4 T cells expressing any of the 3 cytokines measured (i.e. IFNγ, IL2 and TNFα) and expressed as a % of total CD4 population. Open symbols represent SC negative patients (n = 6) and solid symbols represent SC positive patients (n = 12). The horizontal line shows the median. Non-Parametric Mann-Whitney t-test was used for statistical comparisons. (B) Representative flow cytometry dot plots showing the level of expression of IFNγ, IL2 and TNFα after no stimulation (NS), Mtb peptides, PPD and mitogen (MITO) in the whole blood ICS assay. The numbers in the quadrant represent the frequency of cells expressing each cytokine. The gating strategy is shown in Figure S1.

We first compared the overall frequencies of total Mtb- and/or PPD-specific CD4 T cells expressing IFNγ, TNFα or IL2 in patients who were SC positive and SC negative at baseline (Figure 1A). While there was no difference in the magnitude of total mycobacteria-specific CD4 T cells, mitogen responding CD4 T cells were approximately 3.5 fold higher (p = 0.01) in patients who were SC negative at baseline than those who were SC positive (median: 8.7% [IQR: 3.9–11.8] and 2.5% [IQR: 1.7–3.5], respectively). As shown in Table 1, four patients who were SC negative and eight patients who were SC positive at baseline showed detectable cytokine expression (Figure 1A). Figure 1B shows representative dot-plots of CD4 T cells expressing IFNγ, TNFα and IL2 in response to Mtb, PPD and mitogen stimulation in a patient responding to both antigens.

**Figure 2:**
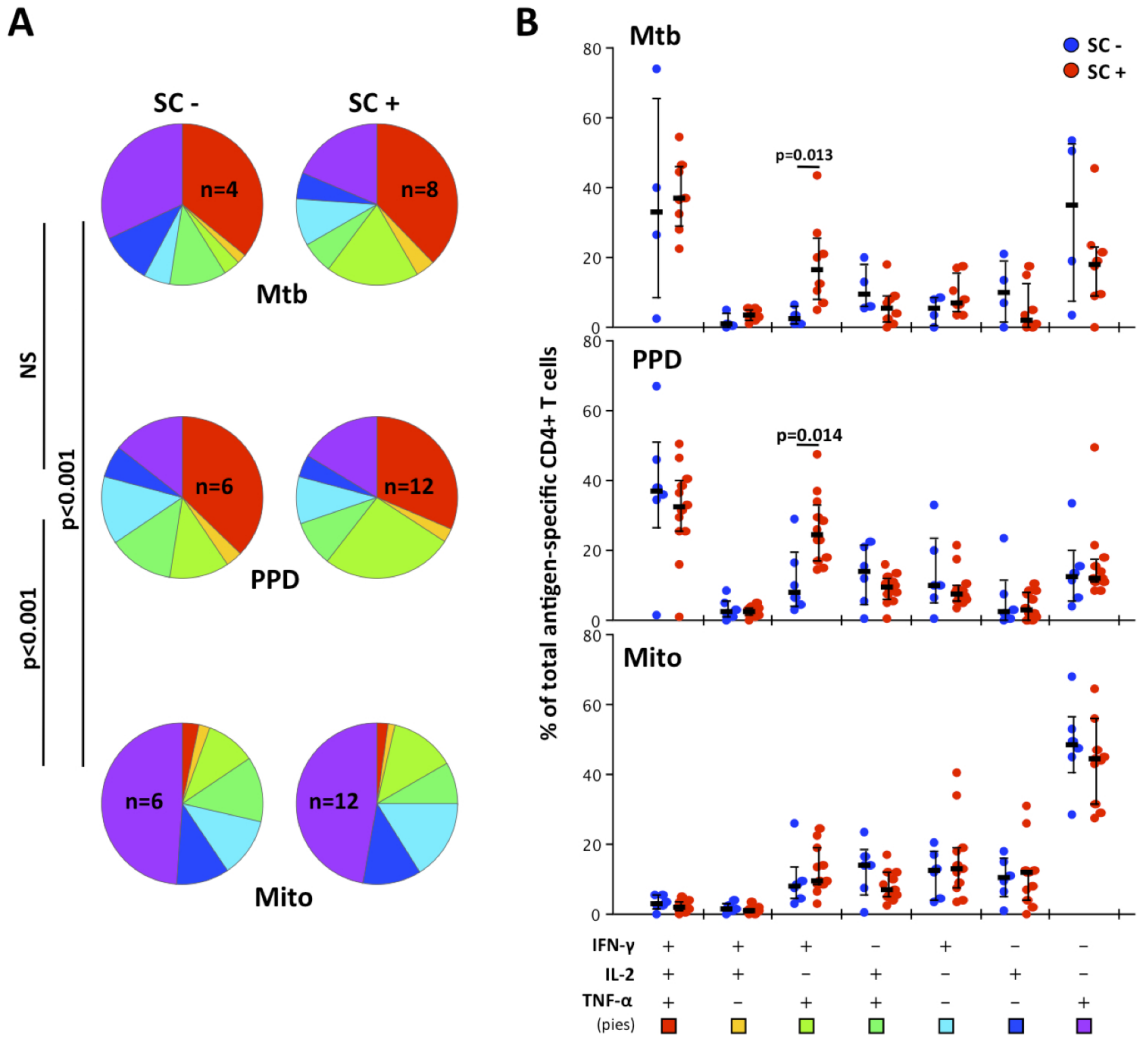
Comparison of the polyfunctional profiles of Mtb-, PPD- and Mito-specific CD4 T cells at baseline between patients who were sputum culture (SC) negative or positive. Pie and Bar charts showing the proportion (A) and relative frequency (B) of each cytokine combination after Mtb, PPD and mitogen (Mito) stimulation. The colors of the pies correspond to the permutation color grid at the foot of 2B, where this corresponds to the proportional frequency of cytokine-expressing combinations. Twelve of 18 patients responded in the ICS assay to the Mtb antigen cocktail: 4 SC negative and 8 SC positive. All 18 patients responded to PPD and Mito stimulations: 6 SC negative and 12 SC positive for each ICS stimulus. Statistical analysis of T cell multi-cytokine expression differences between groups, represented in the pie charts in Figure A, was made using a non-parametric partial permutation method within SPICE [23] and differences between antigen-specific CD4 T cells in Figure B using the student's t-test.

Using Boolean gating to examine multiple cytokine combinations at the single cell level (i.e. polyfunctional responses), we found no differences in the polyfunctional capacity between Mtb, and PPD responsive CD4 T cells. The proportion of patients who responded in a polyfunctional manner to Mtb antigens (n = 12 patients), PPD and mitogen (n = 18 patients) in the ICS assay are shown in Figure 2A. On closer examination, the predominant cytokine combinations in response to Mtb or PPD stimulation were IFNγ^+^IL2^+^TNFα^+^ (±30% of total CD4 T cells) and IFNγ^+^IL2^−^TNFα^+^ (±20%). The proportions of these dual expressing mycobacteria-specific CD4 T cells were significantly higher in SC positive than SC negative patients (p = 0.013 for Mtb and p = 0.014 for PPD, Figure 2B). The response to mitogen stimulation was predominantly mono-functional (±70%), with IFNγ^−^IL2^−^TNFα^+^ (±40%) being most prevalent.

### Mtb- and PPD-specific CD4 T cells show a late differentiated (LD) memory phenotype in patients who are SC positive at baseline

**Figure 3:**
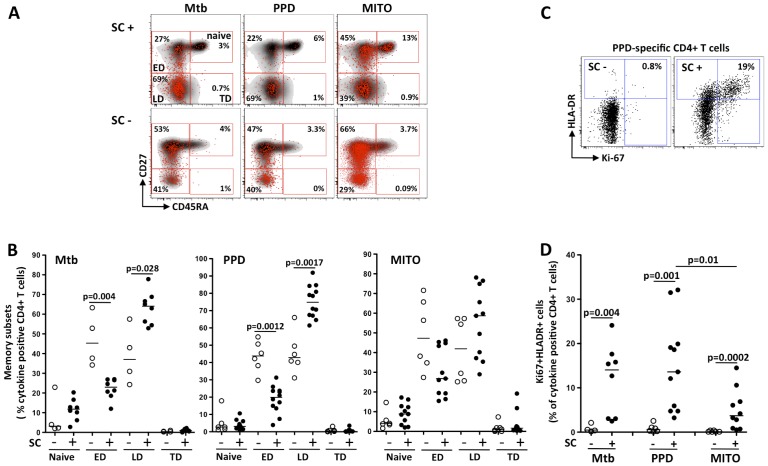
Comparison of memory maturation and activation profiles of antigen-specific CD4 T cells at baseline between sputum culture (SC) negative and positive individuals. (A) Representative overlay flow cytometry dot/density plots of TB-, PPD- and mitogen (mito)- specific CD4 T cell subsets (red) onto total CD4 sub-population (grey) for an individual with positive SC (top panel) and negative SC (bottom panel). Different CD4 subsets are shown as Naïve, Early Differentiated memory (ED), Late Differentiated memory (LD) and Terminally Differentiated (TD). The numbers in each quadrant represent the proportion of antigen-specific cell within each subset (red dots). (B) Proportion of naïve, ED, LD and TD subsets in Mtb (4 SC negative and 8 SC positive), PPD (6 SC negative and 12 SC positive) and mitogen (6 SC negative and 12 SC positive) responsive CD4 T cells. The open symbols represent patients who were SC negative and solid symbols represent patients who were SC positive. The horizontal line corresponds to the median. Non-Parametric Mann-Whitney *t*-test was used for statistical comparisons. (C) Representative flow cytometry dot-plots of the level of expression of Ki-67 and HLA-DR within PPD-specific CD4 T cell in a SC negative and a SC positive representative patient. The numbers represent the proportion of cells co-expressing Ki-67 and HLA-DR. (D) Proportion of activated cells (co-expressing Ki-67 and HLA-DR) in Mtb (4 SC negative and 8 SC positive), PPD (6 SC negative and 12 SC positive) and mitogen (6 SC negative and 12 SC positive) responsive CD4 T cells. Non-Parametric Mann-Whitney t-test was used for statistical comparisons.

We next evaluated the memory differentiation profiles of mycobacteria-specific CD4 T cells at baseline. Using the differentiation markers CD45RA and CD27, we were able to discriminate four CD4 T cell populations. Figure 3A shows representative plots of naïve (CD45RA^+^CD27^+^), early differentiated memory (ED: CD45RA^−^CD27^+^), late differentiated memory (LD: CD45RA^−^CD27^−^) and terminally differentiated cells (TD: CD45RA^+^CD27^−^). Figure 3B shows that there were significantly higher proportions of ED Mtb- and PPD-specific CD4 T cells in individuals who were SC negative compared to those who were SC positive (p = 0.004 and p = 0.0012, respectively). Conversely, there was a higher proportion of LD Mtb- and PPD-specific CD4 T cells in SC positive patients (p = 0.028 and p = 0.0017, respectively). No difference in memory differentiation profiles in mitogen responsive CD4 T cells was observed (Figure 3B).

### Activated and proliferation-competent Mtb- and PPD-specific CD4 T memory cells are enriched in patients who are SC positive at baseline

We compared the relative proportions of activated (HLA-DR^+^) and proliferating (Ki67^+^) antigen-specific CD4 T cells in SC negative and SC positive patients. Figure 3C shows representative flow plots of PPD-specific CD4 T cells co-expressing Ki67 and HLA-DR. Overall, the percentages of Mtb, PPD and mitogen responsive Ki67^+^HLA-DR^+^ CD4 T cells were higher in SC positive than SC negative patients (p = 0.004, p = 0.0009 and p = 0.001, respectively; Figure 3D). When these double positive CD4 T cells were gated (Figure 3C) and analyzed for expression of naïve and memory markers, both Mtb and PPD-specific Ki67^+^HLA-DR^+^ CD4 T cells fell mainly within the LD memory sub-population (Figure S2). Taken together, these data show that distinct subsets of Mtb and PPD-specific CD4 T cells were able to differentiate between SC negative and SC positive patients, and the characteristics of these cells were the dual expression of TNFα and IFNγ, markers of LD stage memory or co-expression of HLA-DR and Ki67.

### Polyfunctionality of Mtb- and PPD-specific CD4 T cells increased during the course of MDR-TB treatment

**Figure 4:**
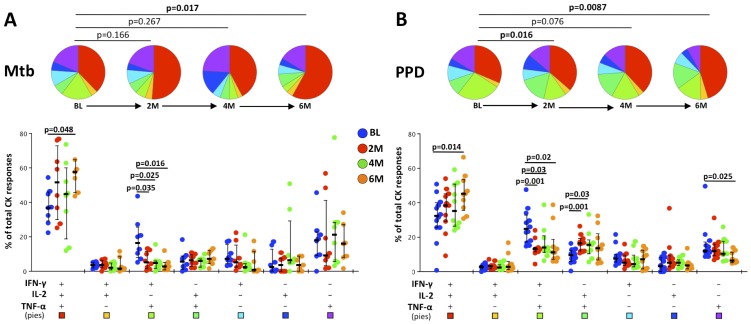
Evolution of the polyfunctional profile of Mtb- and PPD-specific CD4 T cells over time of TB chemotherapy in individuals with positive sputum culture (SC) at baseline. Pie and Bar charts showing changes in the proportions and relative frequency of multi-cytokine combinations after Mtb (A) and PPD (B) stimulation over time from baseline (BL, pre-treatment), 2, 4 and 6 months (M) of treatment. The color codes in the pies correspond to the permutation color blocks at the foot of each bar chart. Eight of the patients who were SC positive at baseline and responded to the Mtb antigen cocktail are shown in A. Twelve of the patients who were SC positive and responded to PPD stimulations and are shown in B. Statistical analysis of the changes in CD4+ T cell multi-cytokine proportions over time, represented in the pie charts, was made using the non-parametric partial permutation method within SPICE [23] and differences between the relative frequency of antigen-specific CD4+ T cells over time, represented by the solid colored symbols in the bar chart, was assessed using the Wilcoxon T-test.

We then analyzed the evolution of mycobacteria-specific CD4 T cell subsets over the course of treatment in patients who were SC positive on admission. Figure 4 shows the proportion of CD4 T cells producing cytokines (IFNγ, IL2 and TNFα) in response to Mtb (Figure 4A) or PPD (Figure 4B) stimulation and changes in these over time of chemotherapy. There was a progressive increase in the polyfunctional capacity of both Mtb- and PPD-specific CD4 T cells (p = 0.017 and p = 0.0087, respectively) between the time of admission and 6 months of treatment. In particular, the proportion of triple-expressing IFNγ^+^IL2^+^TNFα^+^ cells increased from baseline to 6 months of treatment (p = 0.048 and p = 0.014 for Mtb and PPD responses, respectively). In addition, the dual expressing population IFNγ^+^IL2^−^TNFα^+^ of Mtb and PPD-specific CD4 T cells, which discriminated between sputum culture status prior to treatment, significantly decreased within the first 2 months of chemotherapy (p = 0.035 and p = 0.001 in response to Mtb and PPD stimulation respectively) in patients who were SC positive at baseline. There was no change in the polyfunctional nature of mitogen responsive CD4 T cells (data not shown). While there was no significant change to the overall frequency of mycobacteria-specific CD4 T cells over time, the proportion of mitogen responsive CD4 T cells significantly increased as early as 2 months after the initiation of treatment (p = 0.002, Figure S3). Of note, the increase in polyfunctional PPD-specific CD4 T cells at 6 months of chemotherapy was observed in both ED and LD memory populations (p = 0.009 and p = 0.006, respectively), and the LD population was more polyfunctional than the ED memory subset at 6 months (p = 0.001) (data not shown). Additionally, there was a trend toward an increased proportion of ED cells over time of treatment (Figure S4). These findings suggest that polyfunctionality and the stage of memory differentiation are not necessarily associated. Collectively, these data show that effective therapy leads to an increase in the polyfunctional nature of Mtb- and PPD-specific CD4 T cells, regardless of memory status, and induces an increased responsiveness to mitogen stimulation.

### Decreased activation status of Mtb and PPD-specific CD4 T cells during treatment

**Figure 5:**
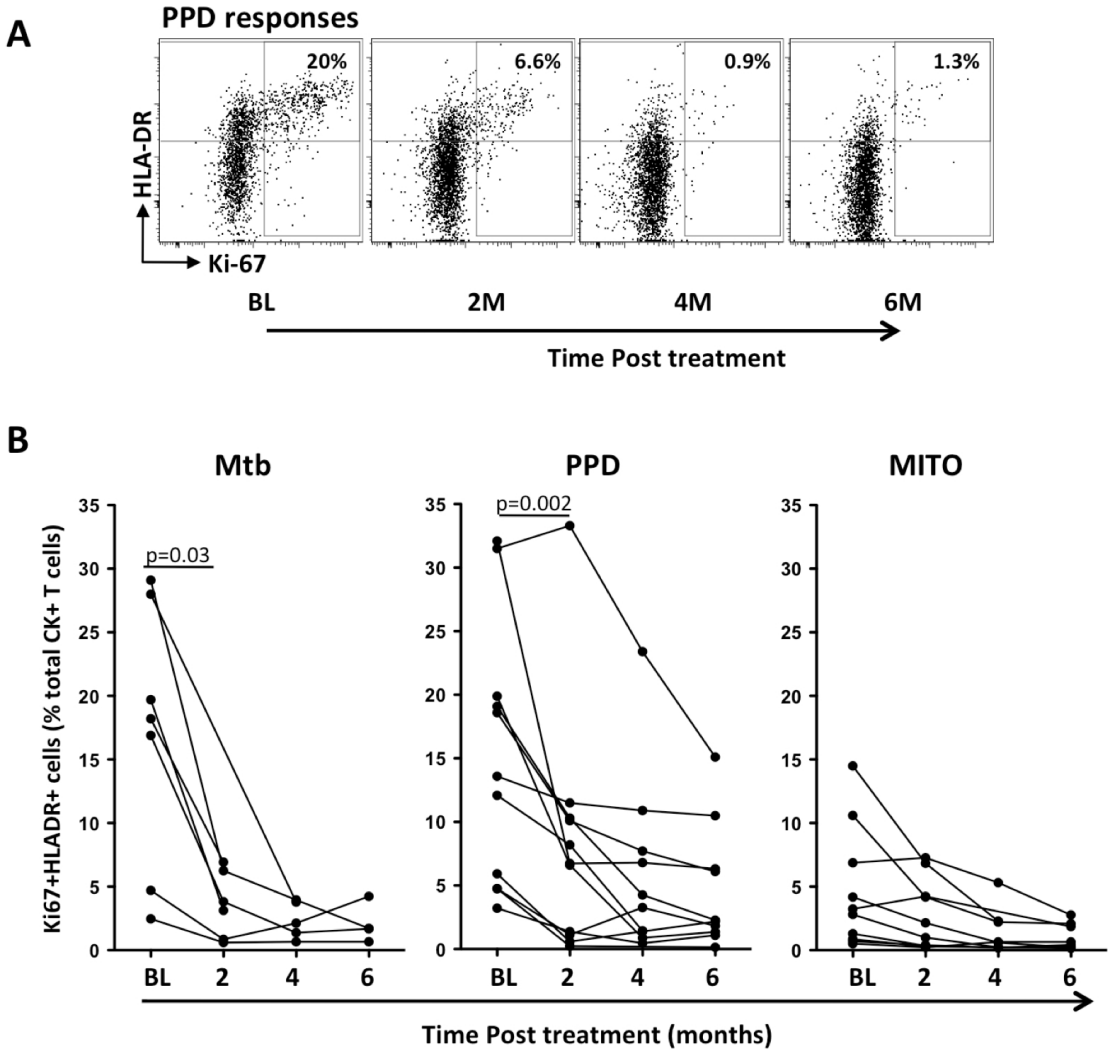
Changes in the proportion of antigen-specific CD4 T cells co-expressing HLA-DR and Ki67 over time of chemotherapy in individuals with positive SC at baseline. (A) Representative flow cytometry dot-plots showing the level of Ki-67 and HLA-DR co-expression within PPD-specific CD4 T cells at baseline (BL), 2, 4 and 6 months (M) of treatment. The numbers in the quadrants represent the proportion of antigen-specific CD4 T cells co-expressing Ki-67 and HLA-DR. (B) Proportion of activated (Ki-67^+^HLA-DR^+^) cells within Mtb, PPD and mitogen responsive CD4 T cells over time (months). The statistical differences were assessed using Wilcoxon matched pairs test.

To correlate changes in the proportions of activated and proliferating mycobacteria-specific CD4 T cells with sputum culture conversion, we determined the frequencies of Ki-67^+^HLA-DR^+^ antigen-specific CD4 T cells over time. Figure 5A shows representative dot plots, where the frequency of Ki-67^+^HLA-DR^+^ PPD-specific CD4 T cells diminished from 20% at baseline to 1.3% at 6 months of treatment. Consolidation of these data in Figure 5B shows a rapid and significant reduction in the proportion of Ki-67^+^HLA-DR^+^ Mtb- and PPD-specific CD4 T cells within the first 2 months of treatment (p = 0.03 and p = 0.002, respectively). No significant changes in the activation profile of mitogen responsive CD4 T cells were observed over time. Collectively, these results show that TB treatment induced rapid changes leading to lower activation and proliferation of mycobacteria-specific CD4 T cells.

### The proportion of activated mycobacteria-specific CD4 T cells at baseline can predict the time to sputum culture conversion

**Figure 6:**
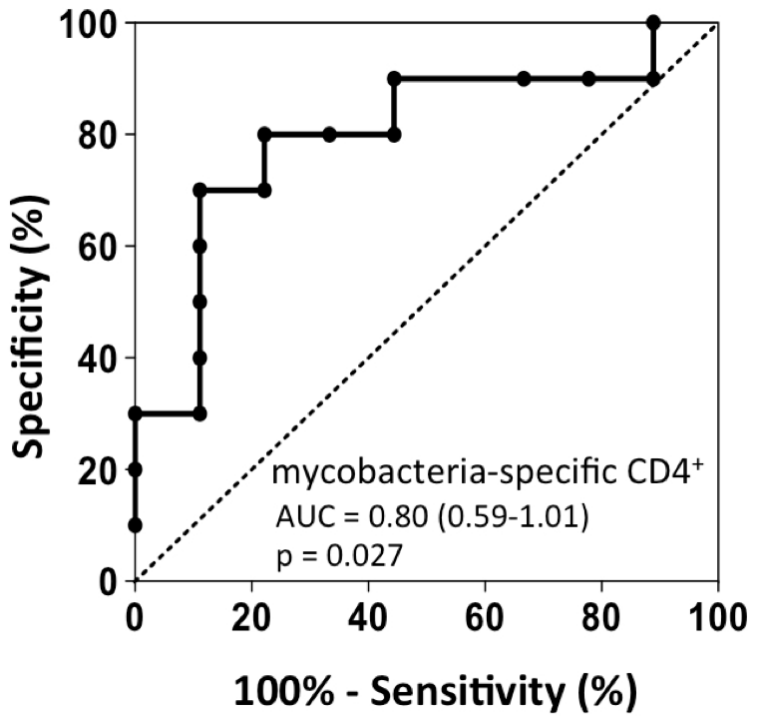
ROC curve analysis of mycobacteria-specific Ki-67^+^HLA-DR^+^ CD4 T cells with time to sputum culture conversion. Receiver Operating Characteristic curve for the proportion of mycobacteria-specific Ki-67^+^HLA-DR^+^ CD4 T cells at baseline with the time to sputum clearance. The area under the curve (AUC), p-value and 95% confidence interval (c.i.) are shown on the graph. The dotted line represents an AUC of 0.5, which would depict a random test.

Longer time to sputum culture conversion (Table 1) was significantly associated with a greater proportion of PPD-specific Ki-67^+^HLA-DR^+^ CD4 T cells present at baseline (r = 0.68, p = 0.025, data not shown). Mtb-specific associations were not significant, although the trend was similar. Receiver operating characteristic (ROC) curves show that the proportion of Ki-67^+^HLA-DR^+^ CD4 T cells at baseline was predictive of time to sputum culture conversion. This was the case when using either PPD or combined PPD/Mtb-specific Ki-67^+^ CD4 T cells (for PPD, AUC of 0.833 (p = 0.068); 95% ci: 0.57–1.098 and for Mtb/PPD, AUC of 0.80; p = 0.027, Figure 6). Of note, neither the frequencies, memory maturation profile nor polyfunctional capacity of mycobacteria-specific CD4 T cells had any significant predictive value to time of culture conversion (data not shown).

## Discussion

We interrogated the phenotypic and functional profile of mycobacteria-specific CD4 T cells in the blood of patients who were sputum culture (SC) positive and SC negative on admission. We hypothesized that a sub-population of these cells may be used as markers of bacillary (antigen) load. Two subsets of late differentiated memory and activated proliferating (Ki67^+^HLA-DR^+^) antigen-specific CD4 T cells were identified that could discriminate between patients with and without sputum culturable bacilli. The activated sub-population of cells in the blood, whether Mtb- or PPD-specific, appeared to mirror antigen load in the lungs, and the baseline frequency significantly associated with the time taken to achieve culture conversion. This was further supported by the finding that the lowest frequencies of activated and proliferating mycobacteria-specific CD4 T cells were found in six of the eighteen patients who were SC negative upon enrollment. Although MDR-TB was detectable in these participants, they were most likely responsive to standard chemotherapy, and this was reflected in the lowest detectable frequency of mycobacteria-specific proliferating CD4 T cells in these patients. These data also suggest that our findings may also be applicable to patients with drug susceptible TB.

This study highlights four main findings, each of which underlines the immune response to TB, but can also be applied to assess sputum culture status or to monitor the efficacy of TB treatment. The first finding was that the degree of mycobacteria-specific CD4 T cell memory differentiation is associated with bacillary load, as indicated by SC positivity, and is in agreement with the results of studies of patients with drug susceptible TB. These reports describe higher LD mycobacterial-specific CD4 T cells relative to ED cells in TB patients who were smear or culture positive prior to treatment and a shift to relatively higher proportions of ED CD4 T with treatment or in individuals with cured TB [15], [24]. In addition, patients with persistently active TB disease who remained SC positive after 6 months of treatment were shown to have low frequencies of antigen-specific CD27^+^CD4 T cells [25]. Collectively, these results suggest that chronically high levels of mycobacterial antigens in active TB lead to the accumulation of LD memory cells.

Our second finding was a larger proportion of proliferating (as measured by Ki-67) and activated (as measured by HLA-DR) Mtb and PPD-specific CD4 T cells in patients who were SC positive compared to SC negative patients. Up-regulation of the nuclear protein, Ki-67, occurs when cells leave the G0 (resting) phase and enter into the G1, S, G2 and M phases of the cell cycle [26]. The increased expression of Ki-67, along with HLA-DR co-expression, supports the presence of systemically activated T cells in patients with SC positive TB and likely elevated bacillary load. Our results appear to be in contrast with a study by Day et al, showing higher proportions of activated proliferating CD4 T cells in individuals with latent TB infection and in patients following TB treatment compared to those with active TB disease [27]. However, in this study, proliferative capacity was evaluated in Oregon Green stained peripheral blood mononuclear cells (PBMC) following 6 days of antigen stimulation. In contrast, our results were obtained from whole blood that was stimulated for 12 hours and stained for the nuclear marker Ki-67, which we consider to be more representative of the in vivo condition. In particular, it may be expected that over 6 days, some CD4+ T cell populations may die in the assay and those that respond and proliferate will likely be central memory cells. We speculate that persistently high levels of TB antigens are enough to drive activation of mycobacteria-specific CD4 T cells to proliferation and propose that these cells reflect antigen load in the host and therefore represent potentially useful biomarkers of the extent of Mtb infection.

The third finding of this study was that polyfunctional mycobacteria-specific CD4 T cells increased over time of TB treatment. This is consistent with other reports from studies of patients with drug susceptible TB [28]. In particular, the decline in IFNγ^+^IL2^−^TNFα^+^ CD4 T cells coincident with an increased population of IFNγ^+^IL2^+^TNFα^+^ CD4 T cells is in agreement with Day et al [27], who investigated changes in relation to bacillary load, as defined by sputum smear acid-fast bacilli (AFB), during treatment. These results may indicate that higher mycobacterial load is associated with impaired IL2 production, which recovers with treatment and bacillary clearance [29]. This hypothesis is supported by several cross-sectional studies showing a higher proportion of mycobacteria-specific IFN^+^IL2^−^TNF^+^ or IFNg^+^IL2^−^ CD4 T cells or lower IL2^+^ CD4 T cells in patients with active TB than in patients after successful treatment [24], [30]–[32]. The discordance of our data with one study showing that polyfunctionality diminished upon TB treatment [33] may be explained by differences in the assay conditions, such as the use of PBMCs instead of whole blood, varying sources of serum, and different antigens used in ex vivo stimulations [34]. We also found that the increased polyfunctionality of CD4 T cells was not necessarily linked with the emergence of ED CD4 T cells, as polyfunctional CD4 T cells increased in both ED and LD populations with response to treatment. Moreover, PPD-specific LD cells were more polyfunctional after 6 months of chemotherapy than ED cells, suggesting that memory differentiation and the ability to express multiple cytokines were not associated. While the increased polyfunctional CD4 T cell population, seen as a protective phenotype in multiple experimental studies [35]–[38], may indicate improved immunity, as suggested by Harari et al [28], this may merely reflect a homeostatic response to lowered mycobacterial antigen load.

Our fourth finding was the reduced frequency of activated proliferating mycobacteria-specific CD4 T cells with bacillary clearance and that baseline activation was predictive of time to culture conversion. We hypothesize that, as bacterial burden is cleared with antibiotic treatment, there is less antigenic stimulation and immunity shifts to a more responsive state. This is consistent with the observed increase in polyfunctionality and was reflected in a trend toward an increased proportion of ED cells over time of treatment (Figure S4). A similar association between antigen load and T cell subsets has been described in the context of HIV viral load during early infection [39] and in response to anti-retroviral treatment [40]. To test this hypothesis, a validation cohort would need to be analyzed.

It is important to note that the present study is a pilot involving a small number of patients, which requires validation through a larger cohort study. While the specific analytic approach used in this study is not practical for wider application, identification of a subset of cells that provides a robust predictor of response to TB treatment can form a foundation for developing a simpler assay. Nonetheless, the association between mycobacteria-specific T cell activation and SC positivity and response to treatment is robust, and, if confirmed, could be highly useful in advancing our understanding of T cell biomarkers in TB treatment.

## Supporting Information

Figure S1 **Gating strategy for the measurement of antigen-specific CD4 T cells.**
(PDF)

Figure S2 **Comparison of the memory maturation profiles of activated (i.e. Ki67^+^HLA-DR^+^: +/+) versus non-activated (i.e. Ki67^−^HLA-DR^−^: −/−) mytobacteria-specific CD4 T cells in individuals with a positive SC at baseline.** (**A**) A representative flow cytometry dot plot of Ki67 and HLA-DR expression levels in PPD-specific CD4 T cells. Cells negative for Ki67 and HLA-DR (non-activated, −/−) and positive for both (activated, +/+) are shown in the bottom left and top right quadrants. (**B**) Distribution of activated (+/+) and non-activated (−/−) PPD- and Mtb-specific CD4 T cells within distinct CD4 subpopulations. Horizontal lines depict the median values and non-Parametric Mann-Whitney *t*-test was used for statistical comparisons.
(PDF)

Figure S3 **Comparison of the frequencies of antigen-specific CD4 T cells in response to Mtb, PPD and Mito stimulation in individuals with a positive SC at baseline, and over time of chemotherapy.** The magnitude of antigen-specific CD4 T cells expressing any of the 4 cytokines measured (i.e. IFNγ, IL2 and TNFα) is expressed as a % of total CD4 population. Each line represents an individual. Measurements have been performed at baseline (0), 2, 4 and 6 months after the initiation of TB-MDR therapy. The statistical differences were assessed using Wilcoxon matched paired test.
(PDF)

Figure S4 **Changes in memory maturation profile of antigen-specific CD4 T cells over time of chemotherapy in individuals with positive SC at baseline.** (**A**) Representative flow cytometry dot-plots of the level of expression of CD45RA and CD27 within PPD-specific CD4 T cells at baseline (0), 2, 4 and 6 months after TB-MDR therapy initiation. The numbers in the quadrants represent the proportion of antigen-specific CD4 T within each sub-population. (**B**) Proportion of early differentiated (ED) cells within Mtb, PPD and mitogen responsive CD4 T cells over time (months). The statistical differences were assessed using Wilcoxon matched paired test.
(PDF)

Table S1 **Diagnosis and treatment characteristics of patients enrolled into the study, showing the days between admission and most recent positive sputum culture, method of diagnosis (drug susceptibility testing (DST), Line Probe Assay (LPA) PCR bands signifying R and I resistance), sputum smear at diagnosis, chest X ray cavitation and disease scores, and post admission drug cocktail regimens.**
(DOCX)
