# Supplementary material for: A Subset of Circulating Blood Mycobacteria-Specific CD4 T Cells Can Predict the Time to Mycobacterium tuberculosis Sputum Culture Conversion
Source: PLoS One. 2014 Jul 21;9(7):e102178. doi: 10.1371/journal.pone.0102178 (PMC4105550; doi:10.1371/journal.pone.0102178)
Supplement: Figure S1 — Gating strategy for the measurement of antigen-specific CD4 T cells. (PDF) [file pone.0102178.s001.pdf]

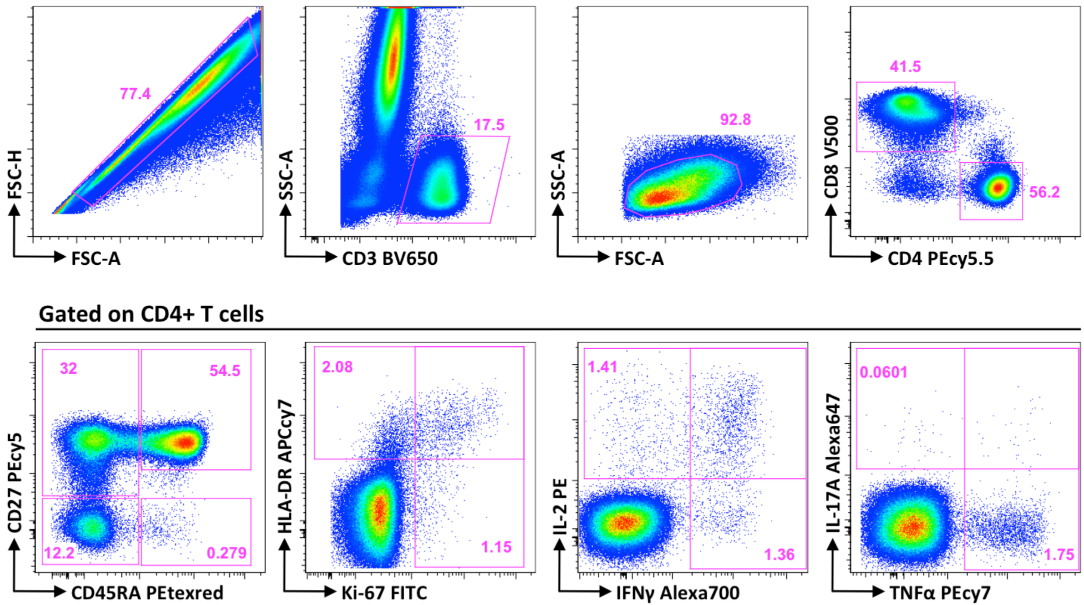

Supplementary Figure 1: Gating strategy for the measurement of antigen-specific CD4 T cells.
