# Supplementary material for: A Subset of Circulating Blood Mycobacteria-Specific CD4 T Cells Can Predict the Time to Mycobacterium tuberculosis Sputum Culture Conversion
Source: PLoS One. 2014 Jul 21;9(7):e102178. doi: 10.1371/journal.pone.0102178 (PMC4105550; doi:10.1371/journal.pone.0102178)
Supplement: Figure S2 — Comparison of the memory maturation profiles of activated (i.e. Ki67+HLA-DR+: +/+) versus non-activated (i.e. Ki67−HLA-DR−: −/−) mytobacteria-specific CD4 T cells in individuals with a positive SC at baseline. (A) A representative flow cytometry dot plot of Ki67 and HLA-DR expression levels in PPD-specific CD4 T cells. Cells negative for Ki67 and HLA-DR (non-activated, −/−) and positive for both (activated, +/+) are shown in the bottom left and top right quadrants. (B) Distribution of activated (+/+) and non-activated (−/−) PPD- and Mtb-specific CD4 T cells within distinct CD4 subpopulations. Horizontal lines depict the median values and non-Parametric Mann-Whitney t-test was used for statistical comparisons. (PDF) [file pone.0102178.s002.pdf]

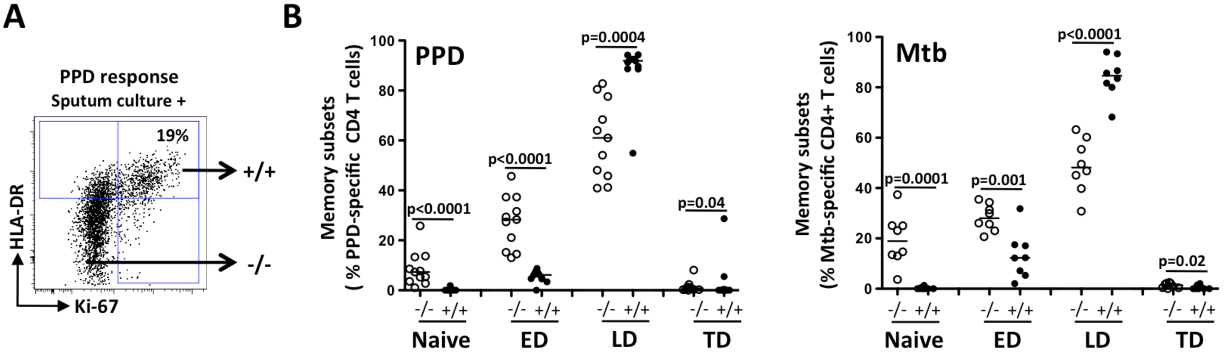

Supplementary figure 2: Comparison of the memory maturation profiles of activated (i.e. Ki67$^+$HLA-DR$^+$: +/+) versus non-activated (i.e. Ki67$^-$HLA-DR$^-$: -/-) mytobacteria-specific CD4 T cells in individuals with a positive SC at baseline. (**A**) A representative flow cytometry dot plot of Ki67 and HLA-DR expression levels in PPD-specific CD4 T cells. Cells negative for Ki67 and HLA-DR (non-activated, -/-) and positive for both (activated, +/+) are shown in the bottom left and top right quadrants. (**B**) Distribution of activated (+/+) and non-activated (-/-) PPD- and Mtb-specific CD4 T cells within distinct CD4 subpopulations. Horizontal lines depict the median values and non-Parametric Mann-Whitney *t*-test was used for statistical comparisons.
