# Supplementary material for: A Subset of Circulating Blood Mycobacteria-Specific CD4 T Cells Can Predict the Time to Mycobacterium tuberculosis Sputum Culture Conversion
Source: PLoS One. 2014 Jul 21;9(7):e102178. doi: 10.1371/journal.pone.0102178 (PMC4105550; doi:10.1371/journal.pone.0102178)
Supplement: Figure S3 — Comparison of the frequencies of antigen-specific CD4 T cells in response to Mtb, PPD and Mito stimulation in individuals with a positive SC at baseline, and over time of chemotherapy. The magnitude of antigen-specific CD4 T cells expressing any of the 4 cytokines measured (i.e. IFNγ, IL2 and TNFα) is expressed as a % of total CD4 population. Each line represents an individual. Measurements have been performed at baseline (0), 2, 4 and 6 months after the initiation of TB-MDR therapy. The statistical differences were assessed using Wilcoxon matched paired test. (PDF) [file pone.0102178.s003.pdf]

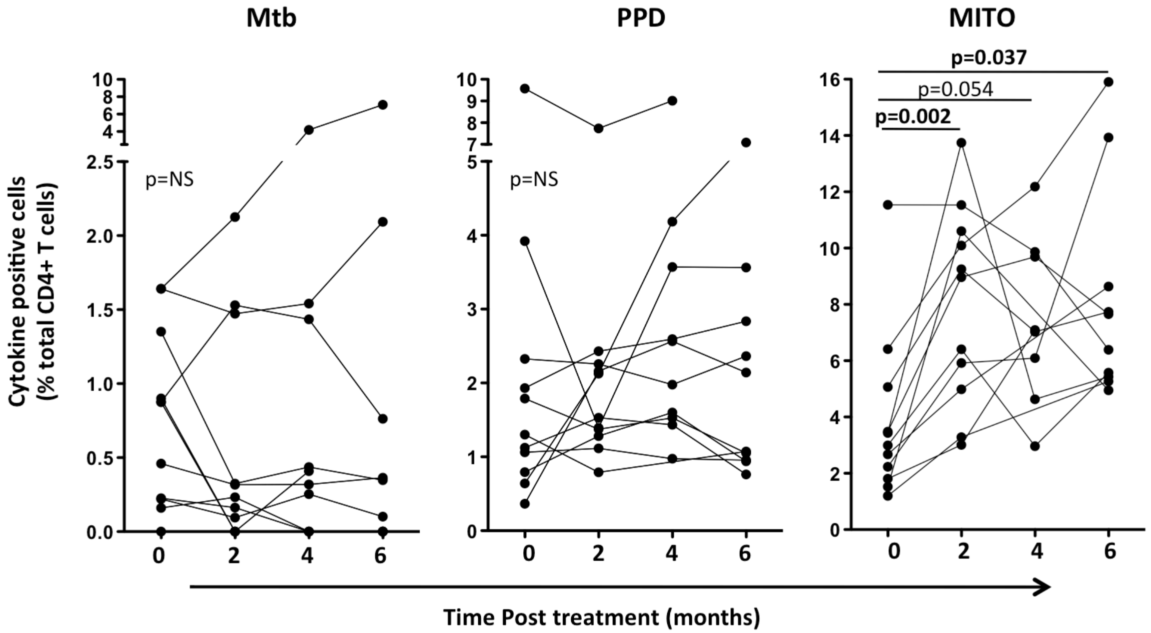

Supplementary figure 3: Comparison of the frequencies of antigen-specific CD4 T cells in response to Mtb, PPD and Mito stimulation in individuals with a positive SC at baseline, and over time of chemotherapy. The magnitude of antigen-specific CD4 T cells expressing any of the 4 cytokines measured (i.e. IFNγ, IL2 and TNFα) is expressed as a % of total CD4 population. Each line represents an individual. Measurements have been performed at baseline (0), 2, 4 and 6 months after the initiation of TB-MDR therapy. The statistical differences were assessed using Wilcoxon matched paired test.
