# Supplementary material for: A Subset of Circulating Blood Mycobacteria-Specific CD4 T Cells Can Predict the Time to Mycobacterium tuberculosis Sputum Culture Conversion
Source: PLoS One. 2014 Jul 21;9(7):e102178. doi: 10.1371/journal.pone.0102178 (PMC4105550; doi:10.1371/journal.pone.0102178)
Supplement: Figure S4 — Changes in memory maturation profile of antigen-specific CD4 T cells over time of chemotherapy in individuals with positive SC at baseline. (A) Representative flow cytometry dot-plots of the level of expression of CD45RA and CD27 within PPD-specific CD4 T cells at baseline (0), 2, 4 and 6 months after TB-MDR therapy initiation. The numbers in the quadrants represent the proportion of antigen-specific CD4 T within each sub-population. (B) Proportion of early differentiated (ED) cells within Mtb, PPD and mitogen responsive CD4 T cells over time (months). The statistical differences were assessed using Wilcoxon matched paired test. (PDF) [file pone.0102178.s004.pdf]

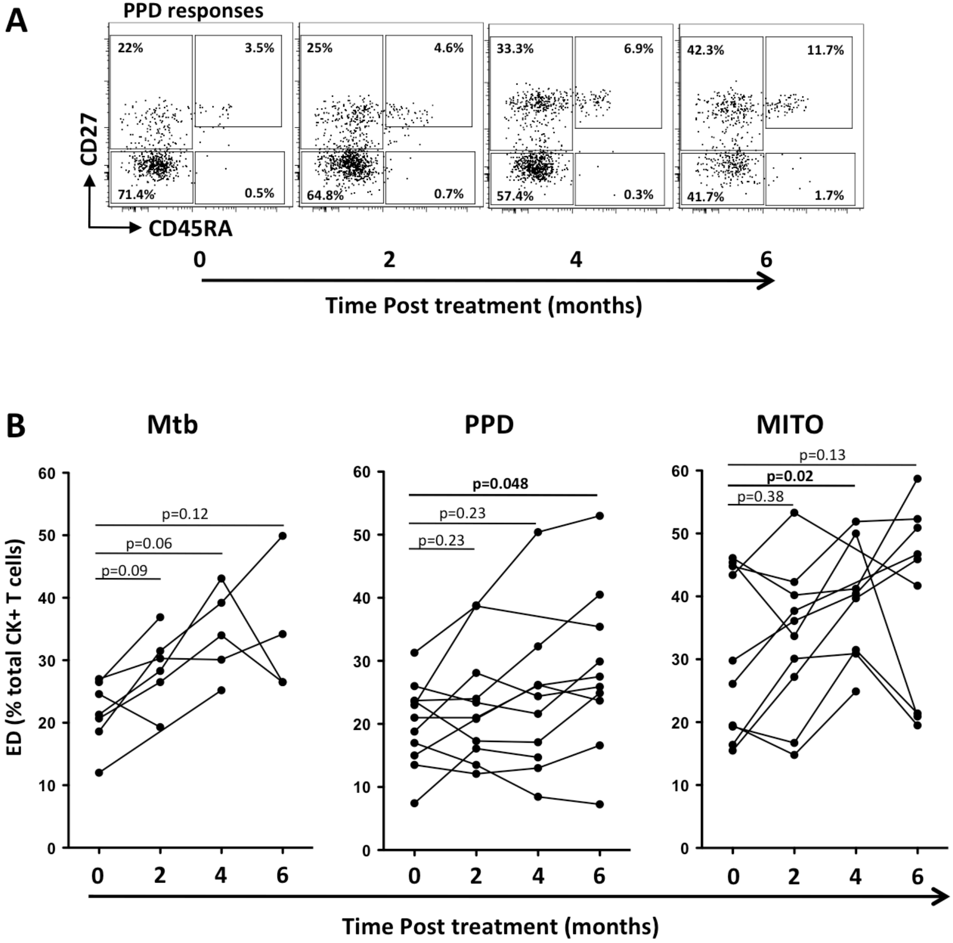

Supplementary figure 4: Changes in memory maturation profile of antigen-specific CD4 T cells over time of chemotherapy in individuals with positive SC at baseline.

(**A**) Representative flow cytometry dot-plots of the level of expression of CD45RA and CD27 within PPD-specific CD4 T cells at baseline (0), 2, 4 and 6 months after TB-MDR therapy initiation. The numbers in the quadrants represent the proportion of antigen-specific CD4 T within each sub-population. (**B**) Proportion of early differentiated (ED) cells within Mtb, PPD and mitogen responsive CD4 T cells over time (months). The statistical differences were assessed using Wilcoxon matched paired test.
