# Supplementary material for: A Subset of Circulating Blood Mycobacteria-Specific CD4 T Cells Can Predict the Time to Mycobacterium tuberculosis Sputum Culture Conversion
Source: PLoS One. 2014 Jul 21;9(7):e102178. doi: 10.1371/journal.pone.0102178 (PMC4105550; doi:10.1371/journal.pone.0102178)
Supplement: Table S1 — Diagnosis and treatment characteristics of patients enrolled into the study, showing the days between admission and most recent positive sputum culture, method of diagnosis (drug susceptibility testing (DST), Line Probe Assay (LPA) PCR bands signifying R and I resistance), sputum smear at diagnosis, chest X ray cavitation and disease scores, and post admission drug cocktail regimens. (DOCX) [file pone.0102178.s005.docx]

**Supplementary Table 1:** Diagnosis and treatment characteristics of patients enrolled into the study, showing the days between admission and most recent positive sputum culture, method of diagnosis (drug susceptibility testing (DST), Line Probe Assay (LPA) PCR bands signifying R and I resistance), sputum smear at diagnosis, chest X ray cavitation and disease scores, and post admission drug cocktail regimens.

| **Pre-admission diagnosis** | | | | | | | |  | **Post admission treatment** | | | | | | | | | | | | | | |
| --- | --- | --- | --- | --- | --- | --- | --- | --- | --- | --- | --- | --- | --- | --- | --- | --- | --- | --- | --- | --- | --- | --- | --- |
|  | **Patient #** | **Days between admission and most recent positive sputum culture** | **Method of MDR diagnosis** | **Sputum smear at MDR diagnosis** | **CXR cavitation score^1^** | **CXR disease score^1^** |  | Kanamycin | Pyrazinamide | Ethambutol | Ofloxacin | Ethionamide | Terizodone | PAS | Clarithromycin | Clofazimine | Capreomycin | Moxifloxacin | HD Isoniazid | Azithromycin | Linezolid | Rifampicin | Amikacin |
|  |  |  |  |  |  |  |  |  |  |  |  |  |  |  |  |  |  |  |  |  |  |  |  |
|  | 1 | 0 | DST | AFB+++ | 0,00 | 7,00 |  | ✓ | ✓ | ✓ | ✓ | ✓ | ✓ |  |  |  |  |  | ✓ |  |  | ✓ |  |
|  | 2 | 0 | PCR | ND | 0,25 | 2,00 |  | ✓ | ✓ | ✓ | ✓ |  | ✓ |  |  |  |  |  |  |  |  |  |  |
|  | 3 | 0 | DST | AFB++ | 3,50 | 7,00 |  | ✓ | ✓ | ✓ | ✓ | ✓ |  |  |  |  |  |  |  |  |  |  |  |
|  | 4 | 0 | DST | AFB+++ | 3,50 | 7,00 |  | ✓ | ✓ | ✓ | ✓ | ✓ |  |  |  |  |  |  |  |  |  |  |  |
|  | 5 | 0 | DST | AFB+ | 0,00 | 9,00 |  | ✓ | ✓ |  | ✓ | ✓ | ✓ | ✓ |  | ✓ |  |  |  | ✓ |  |  |  |
|  | 6 | 0 | DST | AFB+++ | 2,00 | 6,00 |  | ✓ | ✓ | ✓ |  | ✓ | ✓ |  |  |  |  | ✓ |  |  |  |  |  |
|  | 7 | 0 | DST | AFB+ | 1,00 | 8,00 |  | ✓ | ✓ |  | ✓ |  | ✓ | ✓ |  | ✓ |  |  |  | ✓ |  |  |  |
|  | 8 | 0 | DST | AFB+ | 0,00 | 7,00 |  | ✓ |  |  |  |  | ✓ | ✓ |  |  |  | ✓ | ✓ | ✓ |  |  |  |
|  | 9 | 0 | DST | AFB+++ | 1,25 | 8,00 |  | ✓ | ✓ |  | ✓ | ✓ | ✓ | ✓ |  | ✓ |  |  |  | ✓ |  |  |  |
|  | 10 | 0 | DST | AFB+++ | 6,00 | 11,00 |  | ✓ | ✓ | ✓ | ✓ | ✓ | ✓ |  |  |  |  |  |  |  |  |  |  |
|  | 11 | 0 | DST | ND | 1,25 | 7,00 |  | ✓ | ✓ |  |  |  | ✓ | ✓ | ✓ | ✓ | ✓ | ✓ | ✓ | ✓ | ✓ |  |  |
|  | 12 | 0 | DST | AFB+++ | 2,00 | 7,00 |  | ✓ | ✓ |  | ✓ |  | ✓ | ✓ | ✓ | ✓ |  |  | ✓ |  |  |  |  |
|  | 13 | 13 | DST | negative | 0,00 | 1,00 |  | ✓ | ✓ | ✓ | ✓ | ✓ |  |  |  |  |  |  |  |  |  |  |  |
|  | 14 | 72 | PCR | AFB+++ | 0,00 | 6,00 |  | ✓ | ✓ | ✓ | ✓ | ✓ | ✓ |  |  |  |  |  |  |  |  |  |  |
|  | 15 | 53 | PCR | AFB++ | 0,00 | 6,00 |  | ✓ | ✓ | ✓ | ✓ | ✓ | ✓ |  |  |  |  |  |  |  |  |  |  |
|  | 16 | 42 | PCR | negative | 0,00 | 0,00 |  | ✓ | ✓ | ✓ | ✓ | ✓ | ✓ |  |  |  |  |  |  |  |  |  |  |
|  | 17 | 58 | DST | negative | 5,00 | 9,00 |  | ✓ | ✓ | ✓ | ✓ | ✓ |  |  |  |  |  |  |  | ✓ |  |  | ✓ |
|  | 18 | 112 | DST | negative | 0,00 | 5,00 |  | ✓ | ✓ |  | ✓ |  | ✓ | ✓ |  |  |  |  |  | ✓ |  |  |  |
|  |  |  |  |  |  |  |  |  |  |  |  |  |  |  |  |  |  |  |  |  |  |  |  |
|  |  |  |  |  |  |  |  |  |  |  |  |  |  |  |  |  |  |  |  |  |  |  |  |
| 1 | See materials and methods for definition of chest X-ray (CXR) cavitation and disease score | | | | | | | | | | | | | | | | | | | |  |  |  |
|  | HD, High dose | |  |  |  |  |  |  |  |  |  |  |  |  |  |  |  |  |  |  |  |  |  |
|  | ND, not done | |  |  |  |  |  |  |  |  |  |  |  |  |  |  |  |  |  |  |  |  |  |
